# Supplementary material for: Patterns of Intron Gain and Loss in Fungi
Source: PLoS Biol. 2004 Nov 30;2(12):e422. doi: 10.1371/journal.pbio.0020422 (PMC532390; doi:10.1371/journal.pbio.0020422)
Supplement: Table S1 — Also available at http://genes.mit.edu/NielsenEtAl/. (4.3 MB ZIP). [file pbio.0020422.st001.zip › NielsenEtAl/html/316.html]

AN1857.1.NCU09183.1.MG10969.1.FG04829.1


```
 CLUSTAL W (1.82) Multiple Sequence Alignments - Introns Inserted


Sequence 1: NCU09183.1	485 aa
Sequence 2: FG04829.1	491 aa
Sequence 3: MG10969.1	510 aa
Sequence 4: AN1857.1	470 aa
Alignment Length: 522 aa
Number Identitical Residues: 194 aa
Alignment Score (without introns) 10982


MG10969.1 	-MAATQKSLCAI--PADPIEFPADANTLDYARSEDAKCPIRHMREHFIFPTRASLKKKAL
NCU09183.1	MSDFTSKVKVLR--DGQKPEFPSNANTLEYAQSLDAQDELRHFRNEFIIPTRASLKKKAL
FG04829.1 	-MELSSFVERLR--SSAPPKFPSDANSLDFARNLDSQDKLSHLRDEFILPTKKSLKKKAL
AN1857.1  	MSNHVNGVNGVNGVNGVKPVFPENAASKEYAKALDAADPLASFRDKFIIPSKANIQSKRL
          	     .      .  .    ** :* : ::*:  *:   :  :*:.**:*:: .::.* *

MG10969.1 	DGRLP~V1NFELSGPMLDPIA1YPPNHAKPGETATAQN~GTSNTNDDNVTPAVYFCGNSL
NCU09183.1	DGIIP~G1-------------~------------TQAN~GTTTSTDADT-PCIYFVGNSL
FG04829.1 	DGSIP1N~GHAQNG-------~----------SVNGTN~GHTNGQDDDQ-QCIYFVGNSL
AN1857.1  	A----~-~-------------~-------------KPN1-----------PCIYFCGNSL
          	                                     *             .:** ****

MG10969.1 	GLQPKATRDHINAQLETWASIGVHGHFTSWDNSPLKSWQDMAADCAAQSASVVGAS-PDE
NCU09183.1	GAQPKAVRHYLEAQLETWASIGVNGHFSSLSNSPLTPWQDMAADCAAKSAAIVGAADPSE
FG04829.1 	GAQPKAVREHLNAQLETWASVGVNGHFSAMGNSPLPAWQDLAEDCAIRSADIVGAS-PHE
AN1857.1  	GLQPKATAKYMEAHLDTWASIGVNGHFTKIEDSPLDPWWVMAEQAAGSMSKLVGAA-PEE
          	* ****. .:::*:*:****:**:***:   :*** .*  :* :.*   : :***: * *

MG10969.1 	IAIMNTLTANLHFMMASFYRPTEKRHKIISEWKPFPSDT0YAIASQIQWHG-FDTATSLV
NCU09183.1	VVIMNTLTVNLHFMMASFYRPTDKRHKIILEWRPFPSDW~YAFQSQIEWHG-LDPEKSIV
FG04829.1 	IVIMNTLTANLHLLMASFYKPNEKRHKVILEWKPFPSDH~YAIESQVVWHG-LDPEKSMV
AN1857.1  	VIAMGTLTSNLHLLLASFYKPTATKHKILLDWKAFPSDH0YAIESHIAWHDGLDPKKSMV
          	:  *.*** ***:::****:*. .:**:: :*:.****  **: *:: **..:*. .*:*

MG10969.1 	ELHPDE-NYYISTEKILATIDEHAESTALLLLPGIQYWSGQLFDMPLITAHARAKGIVVG
NCU09183.1	EMQPDE-NLYLSTEKILATIDEHAEDAALLLLPGIQYYTGQLFDIPRITKYAQERGIVVG
FG04829.1 	KIEPNE-DHIITTDLILSTIDQHADDTALLLLPGIQYYSGQLFDIPRITAYAQAKGIVVG
AN1857.1  	LIGPDEGEYEIPTQKILSIIDEHADEAALILLPGIQYYTGQYFDINTITEYAHSKGLMVG
          	 : *:*.:  :.*: **: **:**:.:**:*******::** **:  ** :*: :*::**

MG10969.1 	WDLAHAVGNVPLSLHDWDVDFAIWCTYKYLNAGPGAIAGAFVHERHGKVDSD------GF
NCU09183.1	WDLAHCAGNVELQLHDWNVDFAVWCTYKYLNGGPGSMAGAFVHERHGKVDMS--LGKPVF
FG04829.1 	WDLAHAAGNVELKLHDWNVDFACWCTYKYINAGPGSIAGAYVHERHGKVEMNSETGKASY
AN1857.1  	WDLAHAFANVELKLHEWDVDFAVWCTYKYANAGPGSMGGLFVHEKHGKVDYSQGEDSPQF
          	*****. .** *.**:*:**** ****** *.***::.* :***:****: ..  ... :

MG10969.1 	KLRLSGWYGNNKATRFNMAKD~FDPTPGAQGWVVSNPSGIDLASLGAALSVYNLTTPADL
NCU09183.1	KPRLSGWYGADKSVRFNMDKE~FQPTPGAQGFQVSNPSAIDLTSLAAALSVFNKTSMKDL
FG04829.1 	RPRLMGWYGGDKSVRFNMDNN~FIPTAGAGGFQLSNPSAIDLASLSGALSVFNKTTMHDL
AN1857.1  	RHRLAGWYGGDRSVRFKMDNK1FRPIPGAGGFQLSTSSATDLTCLNASLSIFDQTSISEL
          	: ** **** :::.**:* :. * * .** *: :*..*. **:.* .:**::: *:  :*

MG10969.1 	RKKSLWLTAYAEHLLNGILKDEAASSAGDGKKPAFRIITPSNKNERGAQLSVLLREGLLD
NCU09183.1	RSKALVLTAYTEHLLDEIVRRQPE-----GEEPAFKIITPRDPLQRGTQLSLLLRDGLMD
FG04829.1 	RSKALVLTAYSEYLLDQILSES-------GDSELFRIITPRDPLQRGTQLSVLLKDGLLD
AN1857.1  	RRKSVQLTAYLEYLLLKDTTDETR---------PFRIITPSNPEERGAQLSLLLKPGLLQ
          	* *:: **** *:**      ..           *:**** :  :**:***:**: **::

MG10969.1 	VVGEKMEAAGVVCDRRKPDVMRVAPVPMYNSYEDVWRCVDALRKAVMS
NCU09183.1	KVAAALEENGVVCDKRKPNVIRVAPVPMYCRFEDVWKFMEIFEAAIRG
FG04829.1 	NVSAALEENAVICDKRKPGVIRVAPVPLYSRFEDVWRFMQILRGALKN
AN1857.1  	RVADKLQSASIVCDKREPGVVRVAPAPLYNTYSEVWQFVEQLKAAFQE
          	 *.  ::  .::**:*:*.*:****.*:*  :.:**: :: :. *.
```
